# Delay in diagnosis: the experience in Denmark

**DOI:** 10.1038/sj.bjc.6605383

**Published:** 2009-12-03

**Authors:** F Olesen, R P Hansen, P Vedsted

**Affiliations:** 1 The Research Unit for General Practice, Aarhus University, Bartholins Allé 2, Aarhus C DK-8000, Denmark

**Keywords:** diagnosis, delay, Denmark

## Abstract

**Background:**

Denmark has poorer 5-year survival rates than many other Western European countries, and cancer patients tend to have more advanced stages at diagnosis than those in other Scandinavian countries. Part of this may be due to delay in diagnosis. The aim of this paper is to give an overview of the initiatives currently underway to reduce delays.

**Methods:**

Description of Danish actions to reduce delay.

**Results:**

Results of surveys of patient-, doctor- and system-related delays are presented and so are the political initiatives to ensure that cancer is seen as an acute disease.

**Conclusion:**

In future, fast-track diagnosis and treatment will be provided for suspected cancers and access to general diagnostic investigations will be improved. A large national experiment with cancer seen as an acute disease is currently being implemented, and as yet the results are unknown.

The findings from the EUROCARE studies suggest that Denmark, like the United Kingdom, has poorer 5-year survival rates across a range of cancer types than other Western European countries (Sant *et al*, 2001; Karim-Kos *et al*, 2008; Berrino *et al*, 2009; Verdecchia *et al*, 2009). Mortality rates from cancer are also high in Denmark (International Agency for Research on Cancer, 2007). These findings led to a major public debate and a sense of disappointment regarding Danish efforts to control cancer. As a result, a National Cancer Steering Group was established in 1998, chaired by the National Board of Health with representation from all relevant specialties. National Cancer Plans were developed by this steering group in 2000 and 2005, which analysed the possible problems and made recommendations in relation to prevention, diagnosis and treatment.

Cancer incidence in Denmark is relatively high, reflecting lifestyle factors, for example, a relatively high prevalence of smoking (44% of the population in 1987, and 34% in 2000 were daily smokers) (Ekholm *et al*, 2006). As a result, initiatives were launched to reduce smoking, increase exercise, promote healthier diets and reduce excessive exposure to ultraviolet light. A cervical screening programme had been running for several decades. Following the Cancer Plan in 2005, a national breast cancer screening programme was established. In 2008, a decision was made to set up a colorectal cancer screening programme, but this has not yet been implemented. To improve treatment, necessary but politically difficult decisions had to be taken to concentrate cancer-related surgical procedures in fewer hospital centres (Gøtrik and Hansen, 2001).

Another problem to be tackled was that Danish cancer patients seemed to have more advanced stages at diagnosis than those in other Scandinavian countries (Association of the Nordic Cancer Registries, 2007; Berrino *et al*, 2009). This may have been due to bottlenecks at different stages of the clinical pathway with long waits from first symptom to start of treatment. The second Danish National Cancer Plan addressed these issues, recommending pre-planned, well-structured clinical pathways without unnecessary waiting times for investigations and procedures.

This paper provides:

- an overview of the Danish healthcare system to help understand where delays may occur;
- a brief summary of what is known about different phases of delay for cancer patients and
- an outline of the actions currently being undertaken to reduce delays.

## The danish healthcare system

Denmark has a tax-financed healthcare system with free access to medical advice and treatment in general practices and hospitals. All general practitioners (GPs) in Denmark are independent contractors with the public health service (through the regional health authorities) and are remunerated on a mixed fee for service and capitation basis.

Almost all (98%) citizens (Olivarius *et al*, 1997) are registered with a particular general practice, which they have to consult. List size is on average 1550 patients per GP (including children). GPs act as gate-keepers to investigations and hospital referrals. When a patient experiences symptoms, the patient contacts the GP. The GP then decides whether he/she suspects cancer (or other serious illness). The GP may do some simple blood tests, refer the patient for investigations at a hospital (retaining responsibility for the patient) or refer the patient to a hospital department (at which point the patient is no longer the responsibility of the GP).

## Phases of the clinical pathway

**Figure 1:**
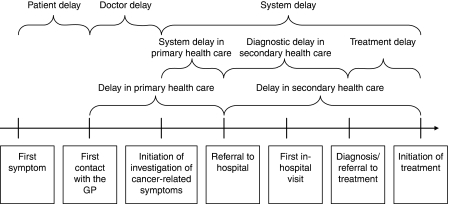
Categorisation of delay.

The pathway from first symptom to treatment can be divided into phases as shown in Figure 1. We defined the three main time periods as patient delay, GP delay and system delay. Specific questions arise in each of these time periods:

- Patient delay – does the patient interpret signs and symptoms appropriately and react in a timely way?
- GP delay – does the GP explore the patient's history appropriately, considering the possibility of cancer?
- System delay – is there a fast and efficient clinical pathway from the moment investigations and/or referral is initiated by the GP until diagnosis is confirmed or rejected and treatment is commenced?

## Evidence related to cancer delays in denmark

**Figure 2:**
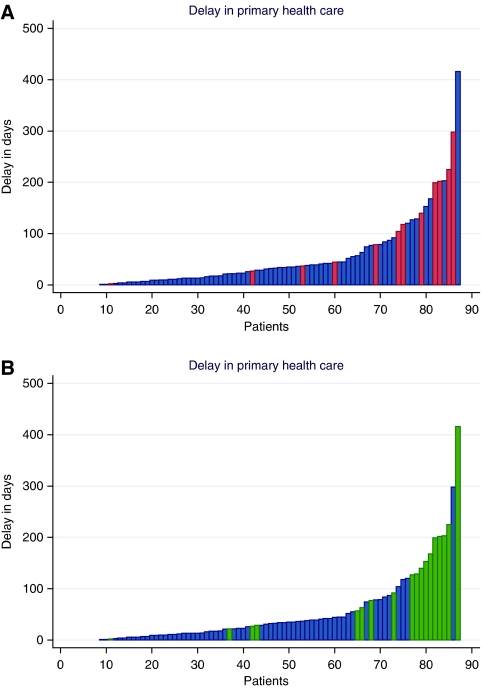
(**A** and **B**) Delay in primary healthcare for 87 consecutive patients in Aarhus County, Denmark with a histologically verified lung cancer diagnosed during two periods of 2003. The patients with the shortest delay are shown in the left part of the *x*-axis and those with the longest delay are shown in the right part of the *x*-axis. Data were based on audit of GP records, referrals, discharge letters and interviews with GPs and patients. Red columns (**A**) are cases where patients presented atypical symptoms without direct suspicion of lung cancer. Green columns (**B**) are cases where a false-negative chest X-ray contributed to the delay. For details see Bjerager (2006).

**Figure 3:**
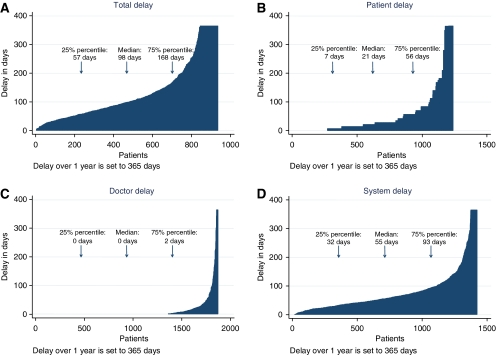
(**A**–**D**) Delay according to questionnaires to GPs for consecutive incident cancers of all types sampled from the regional Hospital Discharge Registry in Aarhus County from 1 September 2004 to 31 August 2005. For definitions of delay see Figure 1. Delay over 1 year is set to 365 days. For details see Hansen (2008).

An analysis of waiting times for 92 patients with lung cancer was undertaken for a PhD thesis (Bjerager, 2006; Bjerager *et al*, 2006). This was based on an audit of medical records, discharge letters and interviews with GPs. This showed that the main contributors to delay were false-negative chest X-rays and non-specific symptoms (Figure 2). Median delay in primary care (i.e. from first contact with a GP to referral to hospital) was 33 days (interquartile interval 12–68 days). These findings raised two questions: is the general clinical pathway too slow, and do we lack appropriate strategies for patients where preliminary investigations are negative and where there are non-specific or uncharacteristic symptoms rather than alarming signs of cancer? A second study undertaken for a PhD thesis raised even more doubts about the organisation of the clinical diagnostic pathway (Hansen, 2008). This thesis was based on patient- and GP-reported delay for more than 2000 consecutive incident cancer patients with all cancer types in the former Aarhus County (population 640 000; with around 3000 new cancer cases per annum). The extent of the different phases of delay is shown in Figure 3 and Table 1. These show that system delay, but also patient delay, is a major contributor to total delay. Further analysis indicated that around half of all cancer patients presented with non-specific or atypical symptoms, which complicated and delayed the diagnostic pathway (data not shown).

## Activities to reduce delays

Activities to reduce cancer delays in Denmark can roughly be divided into two time periods: before and after March 2007, when the data set out above were released at a conference for politicians and decision makers held by the Danish Cancer Society.

During the 1990s, there was increasing general awareness about long hospital waiting times. In response to this, a law was passed in 2001 stating a 2-week waiting time guarantee from diagnosis to treatment. However, there were no guarantees on waiting times for investigations, for example, X-rays ordered by a GP, or for investigations after GP referral to specialists working outside hospitals. Two pre-existing trends were worsened and a new main trend in waiting time patterns followed this initiative. First, we observed a tendency towards ‘double gate-keeping’, where the GP has to refer to a specialist who then gate-keeps again before recommending special investigations (e.g. a computed tomography scan for suspected lung cancer in a patient with a negative chest X-ray). Second, delays of many weeks occurred when a GP requested investigations at a hospital, rather than referring the patient for a specialist opinion at a hospital. Third, delays in hospital from first investigation to final diagnosis increased, as no standard or guarantee had been set for the time from first referral to start of treatment.

In the years following 2000, the problems became more apparent both to clinicians and the lay population. The second Cancer Plan recommended cancer packages with fast track from referral to diagnosis in patients with suspected cancer, but this never became a widespread reality.

Then, during the summer of 2007, following the presentation of the data on different phases of delay, the press presented numerous case stories of unacceptable clinical pathways from symptom to diagnosis. The Danish Cancer Society suggested a new model, making it clear that ‘cancer should be seen as an acute disease’. In the autumn of 2007, the government and Danish Regions (the hospital owners) launched a new diagnostic strategy. The key components of this are:

- Cancer should be dealt with as an acute condition. If the GP or another doctor suspects cancer, only medically necessary waiting times should be accepted in the clinical pathway from symptom to treatment.
- Danish Regions established the service target that a patient should be seen within 2 days following a GP referral with suspicion of cancer.
- Multidisciplinary working groups, chaired by the National Board of Health, were established to describe the ideal clinical pathway for each of the common cancer types. These included maximum acceptable waiting times at each phase of the pathway beginning from the time of referral.
- The government gave the National Board of Health the task of measuring and reporting waiting times.
- A commitment was made to reduce bottlenecks in GP access to diagnostic investigations and to help GPs in difficult cases.
- A commitment was made to invest in necessary equipment.

By spring 2009, the multidisciplinary groups had described fast track referrals for diagnosis and treatment of the common cancers. Strategies are being developed for managing the many patients without a clear-cut initial suspicion of a specific cancer. Substantial investments in diagnostic and therapeutic hardware are currently being made. All regions are considering ways to ensure sufficient support for GPs.

We still lack exact figures for the effect of all these efforts. However, preliminary data from Danish Regions, from The National Board of Health and from independent research institutions show reductions in delays. It is, however, too early to predict final data on reduction in delay in a steady state situation when all the efforts have been implemented. Most importantly, optimism among professionals and lay people is increasing with regard to the prospects of achieving medical excellence.

Initiatives to improve continuing medical education (CME) for GPs have also been undertaken, though surprisingly a survey showed no relation between GP-related doctor delay and the amount of CME undertaken in the years before the survey (Hansen, 2008).

As no formal initiatives have yet been undertaken to reduce patient delay, though the considerable public debate may have raised awareness, systematic research into patient and doctor delay is urgently needed. The Danish Cancer Society and the Novo Nordisk Foundation have recently announced a 30 million DKK (£4 million GBP) grant for further research into the period from first symptom to diagnosis. The government is now under pressure to put public money into this area of research, and in spring 2009, the Department of Health announced its intention to launch a Cancer Plan III in 2010 with anticipated substantial initiatives for awareness and early detection.

## Conclusion

In conclusion, a large national experiment to reduce delays and thereby improve survival rates is now underway. As yet, the results are unknown. However, there is a general impression that the efforts made have increased public satisfaction with the healthcare system's management of serious disease. There is also an increasing political awareness that waiting times are unacceptable to patients who present with symptoms that might be due to serious disease.

Future research into the reasons for different types of delay for each cancer type is urgently needed. In particular, this should focus on the ‘tail’ of delay where patients have very long delays (see Figures 2 and 3). Such research should guide improvement of clinical pathways to provide insights for provision of seamless cancer pathways by publicly funded healthcare systems.

## Figures and Tables

**Table 1:** Delay in days and specification of proportion (%) of patients with delay >3, 6 and 12 months for consecutive incident cancers of all types sampled from the regional Hospital Discharge Registry in Aarhus County from 1 September 2004 to 31 August 2005

	**Patient delay**	**Doctor delay**	**System delay**	**Total delay**
Median (IQI)	21 days (7–56 days)	0 days (0–2 days)	55 days (32–93 days)	98 days (57–168 days)
% of patients with delay >3 months	15.3%	3.5%	25.8%	53.7%
% of patients with delay >6 months	6.8%	1.6%	7.5%	21.5%
% of patients with delay >1 year	2.8%	0.5%	2.6%	8.7%

Abbreviation: IQI=interquartile interval.
